# Belantamab Mafodotin Monotherapy for Multiply‐Relapsed Myeloma: A Retrospective Study From the United Kingdom and the Republic of Ireland

**DOI:** 10.1002/jha2.70039

**Published:** 2025-04-30

**Authors:** Edmund C. R. Watson, Faouzi Djebbari, Fotios Panitsas, Grant Vallance, Samir Asher, Malahat Saeed, Mairi Walker, Matthew Powell, Alexandros Rampotas, Heather Leary, Akhil Khera, Angharad Atkinson, Ni Ni Aung, Gillian Brearton, Joseph Froggatt, Ezzat El Hassadi, Ellen Gokkel, Sarah Lawless, Beena Salhan, Salim Shafeek, Anand Lokare, Carol Stirling, Udo Oppermann, Richard Soutar, Rakesh Popat, Charalampia Kyriakou, Karthik Ramasamy

**Affiliations:** ^1^ Nuffield Department of Orthopaedics, Rheumatology and Musculoskeletal Sciences, Medical Sciences Division University of Oxford Oxford UK; ^2^ Oxford University Hospitals NHS Foundation Trust Oxford UK; ^3^ General Hospital of Athens “LAIKO” Athens Greece; ^4^ University College London Hospitals NHS Foundation Trust London UK; ^5^ University Hospitals Birmingham NHS Foundation Trust Birmingham UK; ^6^ Beatson West of Scotland Cancer Centre Glasgow UK; ^7^ University Hospital of Wales Cardiff UK; ^8^ North Tees and Hartlepool NHS Foundation Trust Hartlepool UK; ^9^ The Clatterbridge Cancer Centre Bebington UK; ^10^ Manchester University NHS Foundation Trust Manchester UK; ^11^ University Hospital Waterford Waterford Ireland; ^12^ Belfast City Hospital Belfast UK; ^13^ Worcestershire Acute Hospitals NHS Trust Worcester UK; ^14^ NHS Greater Glasgow and Clyde Glasgow UK

**Keywords:** BCMA, belantamab mafodotin, immunotherapy, keratopathy, real‐world

## Abstract

**Introduction:**

Belantamab mafodotin (belamaf) was the first BCMA‐targeting immunotherapy licensed in myeloma and was available as monotherapy for a fifth or greater line of treatment. Outcomes for patients in the United Kingdom and the Republic of Ireland potentially differ from those of other regions and may illuminate factors predicting response to therapy.

**Methods and Results:**

We performed a retrospective study of patients treated with belamaf monotherapy in the United Kingdom and the Republic of Ireland. In our cohort of 88 patients, we saw an overall response rate (ORR) of 60%, a median progression‐free survival (PFS) of 8.7 months and a median duration of response (DoR) of 15.8 months. The spectrum of adverse events was as expected, with 84% (71/85) of patients experiencing toxicity. Eye‐related adverse events were the most common, affecting 66% (56/85), leading to dose reduction or delay in 41% (35/85) and discontinuation in 6% (5/85). We specifically assessed physician decision‐making in the context of ocular side effects and found a relatively high frequency of the drug being administered despite moderate levels of toxicity.

**Conclusion:**

Our cohort's ORR is significantly different from those of the DREAMM‐2 and ‐3 trials and other real‐world studies, though a long‐duration response has been reported in other cohorts. Comparative analysis with other real‐world studies did not reveal any significant factors predictive of ORR. The frequent administration of belamaf to patients with eye disease may well reflect a more pragmatic approach than was originally prescribed in the landmark trials.

**Trial Registration:**

The authors have confirmed clinical trial registration is not needed for this submission.

## 1 Introduction

Belantamab mafodotin (belamaf) was the first B cell maturation antigen (BCMA)‐targeting therapy to be licensed for myeloma. BCMA (CD269, encoded by *TNFSRF17*) is a member of the TNF receptor superfamily that signals in response to APRIL (and, to a lesser degree, BAFF) in the bone marrow milieu to promote survival of the plasma cell via NF‐κB activity. It is expressed almost exclusively by plasma cells and appears to be up‐regulated in myeloma—making it a particularly attractive target for immunotherapy [1, 2].

Belamaf comprises an afucosylated IgG1 antibody (belantamab) conjugated by a maleimidocaproyl linker to monomethylauristatin F (mafodotin) [3]. It triggers plasma cell death by mafodotin‐induced apoptosis and Fc‐mediated recruitment of host immune mechanisms [4]. In the landmark DREAMM‐2 study, in 97 patients with multiply relapsed myeloma (median of seven prior lines of therapy), the overall response rate (ORR) was 31% and the median progression‐free survival (PFS) was 2.8 months [5]; where patients responded, the average duration of response (DoR) was 12.5 months [6]. The toxicity profile was dominated by ophthalmological adverse events (AEs)—particularly keratopathy, as well as dry eye and reduced visual acuity—as well as thrombocytopenia [5].

The outcomes were deemed to justify conditional authorisation by the FDA and EMA in 2020, and belamaf monotherapy was subsequently provided in the UK by GSK under a named‐patient programme. However, belamaf then failed to meet its primary endpoint of PFS superiority over pomalidomide with dexamethasone in the DREAMM‐3 trial [7], leading to the non‐renewal of these licences in 2023. Even before the DREAMM‐3 result, belamaf's efficacy as a BCMA‐targeting agent had been overshadowed by ORRs from BCMA‐targeting CAR T and bispecific antibodies (bsAbs) in non‐randomised Phase 2 trials [8, 9, 10]. However, belamaf, as a component of combination therapy in the DREAMM‐7 and DREAMM‐8 trials, showed significant PFS superiority over standard‐of‐care in first‐ or later‐relapse MM [11, 12]. Furthermore, it carries some advantages over CAR T and bsAbs with regard to convenience of administration and toxicity profile.

During the window where belamaf monotherapy was accessible, outcomes for “real‐world” cohorts of patients from various study groups across the United States and Europe were reported. Such studies are critical to understanding a drug's activity in the real‐world context, which is to say, a different context from that of the clinical trial environment. In myeloma clinical trials, patients are typically younger and often have different disease courses up to the treatment start date compared to patients included in real‐world studies of the same drug [13]. More generally, clinical trial patients tend to be less comorbid and subject to less polypharmacy [14]. These differences set up a selection bias with confounding factors of unknown significance that can limit the extrapolation of trial results to the clinic.

Encouragingly, the results from real‐world belamaf cohorts to date broadly mirror those of DREAMM‐2 and DREAMM‐3, including meaningful DoR values that are not reflected by the median PFS (see Table 1). The unanswered question throughout all these reports is how a good response to belamaf can be predicted.

**TABLE 1:** Belamaf‐treated patient cohorts in the literature.

Group	*n*	Prior lines	ORR (%)	FU (months)	PFS (months)	OS (months)	DoR (months)
DREAMM‐2 [6]	97	7	32.0	12.5	2.8	15.3	12.5
DREAMM‐3 [7]	218	4	41.0	11.5	11.2	21.2	NR
ALFA [15]	184	5	32.7	7.8	2.4	8.8	13.1
USA [16]	137	—	30.2	—	5.4	—	—
Spanish [17]	126	5	41.3	13.0	3.5	11.1	—
IFM [18]	106	5	38.1	—	3.5	9.3	—
Israeli [19]	106	6	45.5	11.9	4.7	14.5	8.1
MSK [20]	82	6	45.0	—	6.0	—	11.0
Italian [21]	67	6	31.0	12.0	3.7	12.8	13.8
MD Anderson [22]	39	7	27.0	10.1	1.8	9.2	—
Mayo [23]	36	8	33.0	6.0	2.0	6.5	5.0
Campania [24]	28	6	40.0	6.5	3.0	8.0	—
Greek [25]	27	5	52.0	—	2.0	89.0	12.0

*Note*: Prior lines, FU, PFS, OS and DoR values quoted are median values.

Abbreviations: DoR, duration of response; FU, follow‐up; NR, not reached; ORR, overall response rate; OS, overall survival; PFS, progression‐free survival; Prior lines, number of prior lines of therapy.

We here report the largest “real‐world” study of belamaf monotherapy in the UK and Ireland. The rationale for this study, given the relative abundance of similar reports and now a historical fact of belamaf monotherapy, was as follows. Principally, the treatment pathways of United Kingdom and Republic of Ireland (ROI) patients are likely to be different from those of other countries, which might impact outcomes and help shed light on predictive factors for response. This could inform our understanding of belamaf's activity in triplet combination going forward. Additionally, we designed the study to characterise ophthalmological outcomes in detail and so provide a unique insight into “real‐world” decision‐making regarding belamaf administration—an insight that we expect will prove a useful resource for belamaf in triplet therapy also.

## 2 Materials and Methods

### 2.1 Study Design

This was a retrospective study conducted in the UK and ROI that aimed to capture the real‐world decision‐making and outcomes associated with belamaf monotherapy. All patient cases were eligible for submission, provided the patient had received at least one dose of belamaf monotherapy under the named patient programme. Belamaf was administered according to the summary of product characteristics, which recommends a starting dose of 2.5 mg per kg to be administered intravenously every 3 weeks.

### 2.2 Study Objectives

Given the variety of treatment pathways that lead to fifth or higher‐line therapy and the interacting effect of regional variation with these pathways, we were determined to assess the efficacy of belamaf in patients treated in the UK and ROI. The primary outcome was ORR, where the response for each patient was judged and reported by individual contributors according to the IMWG 2016 guidance [26]. Secondary outcomes included PFS, DoR, overall survival (OS) and AEs.

### 2.3 Data Collection

Patients’ routine clinical records were used to answer questions organised over eight separate modules (see supporting information  for a PDF rendering of the tool). The initial data collection period ran from January 2022 to June 2022 and through to March 2023 for one centre. Contributors were encouraged to provide data for all patients who had commenced belamaf monotherapy at the centre up to the end of the data collection period in an unbiased fashion. Follow‐up data were acquired in September 2023 in those patients whose belamaf had been ongoing at the end of the first period. Additionally, one centre that had continued to collect data on new patients provided these in January 2024. Individual contributors were responsible for interpreting responses to therapy and for grading the severity of AEs using CTCAE criteria and KVA (the Keratopathy and Visual Acuity scale, developed during the DREAMM‐2 study [27]) scores where available for ocular toxicity.

### 2.4 Analysis

Baseline data are presented as medians with interquartile ranges (IQRs) if the features are continuous or as the frequencies with percentages if they are categorical; where a dataset for a given categorical feature is not complete, only the known cohort is used for the denominator.

The Kaplan–Meier method was used for survival analysis. PFS was defined as the time from belamaf start to progression/relapse or death of any cause; OS as the time to death of any cause; DoR as the time from the start of achieving a response until relapse or death. Where patients were still alive and/or on treatment, the censor date was taken as the date of the last review. The Exact method was used to determine ORR confidence intervals for patient subgroups. Fisher's exact test was applied for predicting ophthalmological outcomes given pre‐existing eye conditions.

Data were compiled on Microsoft Excel worksheets. Data cleaning and summary statistics production was performed in Microsoft Excel or using the tidyverse suite of packages for R [28]. Survival analysis was conducted in Stata (StataCorp LLC).

### 2.5 Ethics

This project was conducted under the auspices of service provision, and ethical approval was not sought, as with previous similar studies [29]. The data collection form requested pseudonymous data and was hosted by OpenClinica Community Edition [30].

## 3 Results

### 3.1 Data Entry

**FIGURE 1:**
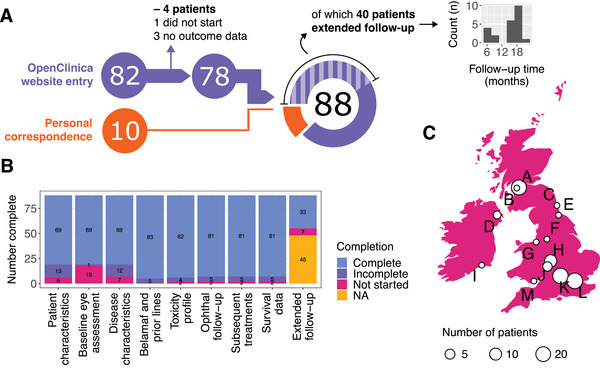
Study format. (A) Completion rates of the various domains of the online data submission form for the 88 patients taken forward for analysis. Note that 10 of these responses were sent directly to the first author during manuscript preparation in the same format as the online form. (B) Distribution of cases across the UK and ROI, with relative *n* numbers indicated. (C) Histogram of the time intervals for the 33 cases where extended follow‐up data were obtained.

Data from 92 patients were submitted from thirteen hospital trusts across the UK and ROI. 82/92 patients were registered via the OpenClinica website during an initial round of data collection; then, for patients continuing belamaf at the end of this first round, contributors were contacted to provide updated clinical outcomes after a median of 16.8 months (IQR 11.6, 19.2). The remaining 10/92 cases were sent electronically in pseudonymised form. Amongst the total cases, three had no outcome data at all and were unusable, and one patient did not start belamaf owing to progressive disease (PD). A total of 88/92 cases were therefore carried forward for analysis. The provenance and quality of data are illustrated in Figure 1.

### 3.2 Baseline Data

Baseline data are shown in Table 2. Ours was a relatively young cohort, with a median age of 65 and modal ECOG and Charlson Comorbidity Index scores of 1 and 0–2, respectively.

**TABLE 2:** Baseline data.

Figure in brackets represents % unless otherwise specified					
		UK and ROI		DREAMM‐2	
**Patient demographics and status**					
Age	Median (IQR)	65	(58, 72)	65	(60, 70)
Gender	Female	36/86	(42)	46/97	(47)
ECOG score	0	14/64	(22)	—	—
	1	31/64	(48)	—	—
	2	15/64	(23)	—	—
	3	3/64	(5)	—	—
	4	1/64	(2)	—	—
Charlson comorbidity index	0–2	35/59	(59)	—	—
	3–4	20/59	(34)	—	—
	≥ 5	4/59	(7)	—	—
**Patient laboratory parameters**					
eGFR (mL/min/1.73 m^2^)	≥ 60	40/67	(60)	67/93	(72)
	30–59	20/67	(30)	24/93	(26)
	< 30	7/67	(10)	2/93	(2)
Anaemia	Present	58/68	(85)	—	—
Lymphopenia	Present	32/68	(47)	—	—
Hypercalcaemia	Present	9/68	(13)	—	—
Neutropenia	Present	19/68	(28)	—	—
Thrombocytopenia	Present	31/68	(46)	—	—
**Disease characteristics**					
Myeloma subtype	IgG	40/81	(49)	65/97	(67)
	IgA	11/81	(14)	32/97	(33)
	IgM	1/81	(1)		
	IgD	2/81	(2)		
	Light chain	23/81	(28)		
	Oligosecretory	3/81	(4)		
	Non‐secretory	1/81	(1)		
Amyloidosis	Present	6/80	(8)	—	—
Plasma cell leukemia	Present	0/79	(0)	—	—
Extramedullary disease	Present	20/80	(25)	22/97	(23)
Cytogenetics	Standard risk	14/42	(33)	—	—
	High risk^a^	28/42	(66)	41/97	(42)
ISS stage	I	13/58	(22)	21/96	(22)
	II	29/58	(50)	33/96	(34)
	III	16/58	(28)	42/96	(43)
R‐ISS stage	I	4/32	(13)	—	—
	II	20/32	(63)	—	—
	III	9/32	(25)	—	—
**Previous treatments**					
Diagnosis to belamaf (years)	Median (IQR)	6.1	(3.7, 8.7)	5.5	(4.0, 7.0)
Number of lines	Median (IQR)	5	(4, 6)	6	—
Treatment	PI	85/87	(98)	—	—
	Bortezomib	79/87	(91)	95/97	(98)
	Carfilzomib	28/87	(32)	74/97	(76)
	IMiD	84/87	(97)	—	—
	Lenalidomide	81/87	(93)	97/97	(100)
	Pomalidomide	73/87	(84)	89/97	(92)
	CD38	79/87	(91)	—	—
	Daratumumab	63/87	(72)	97/97	(100)
	Isatuximab	19/87	(22)	3/97	(3)
	Auto‐HSCT	65/86	(76)	—	—
	Alkylator	71/87	(82)	—	—
	Triple‐class exposed	75/87	(86)	97/97	(100)

^a^ High‐risk cytogenetics as defined by the IMWG [31].

**FIGURE 2:**
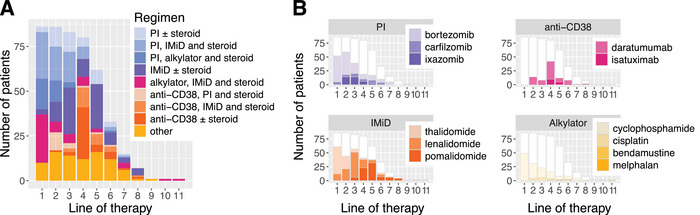
Drug exposure. (A) Summary of regimens at each line of therapy. (B) Drug exposure broken down by class.

Prior treatment data were available for 87/88 patients. Figure 2 conveys the pattern of use. Cyclophosphamide was frequently used in first‐line therapy (49 instances, vs. 49 for thalidomide and 47 for bortezomib); this high relative incidence may reflect an idiosyncrasy of the UK and ROI treatment systems. A total of 86% of patients were triple‐class exposed by the time they commenced belamaf—the granularity of data collection was insufficient to confirm prior drug refractoriness. In total, 9% of patients had not seen an anti‐CD38 agent, contrary to belamaf's eligibility criteria.

### 3.3 Response and Survival Data

**FIGURE 3:**
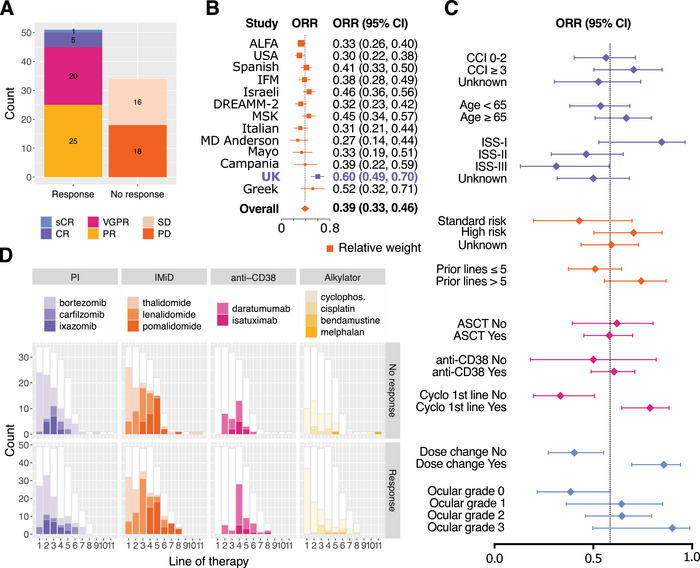
Overall response. (A) Breakdown of overall response. (B) Forest plot for metaregression of ORRs across “real‐world” and DREAMM‐2 trials, showing heterogeneity of results with the current cohort outlying. (C) Subgroup analysis shows a lack of predictive factors by exact method, with the possible exception of ocular toxicity, though small numbers and confounding factors limit analysis. (D) Breakdown of drug exposure, as grouped by response. Cyclophosphamide at the first line was enriched in the responder group.

Belamaf administration data were complete for 73/88 individuals. Patients received a median of four cycles, with the median dose being 2.5 mg/kg and a median cumulative dose of 8.725 mg/kg. Response data were available for 85/88 patients and are summarised in Figure 3. ORR was 51/85 (60%), with VGPR or higher in 26/85 (31%). 16/85 patients had stable disease (SD) and 18/85 had PD. Responders and non‐responders had the same median dose of 2.5 mg/kg; responders received 4.5 cycles and non‐responders 3, with median cumulative doses of 14.3 and 9.3 mg/kg, respectively; these were significantly different by the Student *t*‐test (*p* = 0.027).

Predictors of response were sought and are shown in Figure 3. Preliminary data visualisation demonstrated a possible signal in early exposure to cyclophosphamide (Figure 3D): given in the first line, this associates significantly with the response at an odds ratio (OR) of 5.6 compared to those who do not receive it (Fisher's exact test; *p* value 0.0001; 95% CI, 2.3–20.3). This OR remains at 5.6 when performing the analysis in high cytogenetic risk patients only, though it is no longer statistically significant (*p* value of 0.09; 95% CI, 0.7–73.1). We examined the proximal prior line of therapy to see if we could find an immune priming signal; pomalidomide exposure was highly enriched (used for 45/87 patients, mainly as pomalidomide–dexamethasone [18/45] and isatuximab–pomalidomide–dexamethasone (16/45]); but there was no differential exposure between responders and non‐responders by Fisher's exact test. We compared our response rates with those of other real‐world studies (Figure 3) and confirm that there is heterogeneity across these datasets where our cohort represents the outlier. None of the age, percentage high risk, median prior lines or time to belamaf start explain this variability (Figure S1). Of note, there was no statistically significant difference in response between the three categories of renal impairment (as described in Table 2), with a *p* value of 1 by Fisher's exact test.

**FIGURE 4:**
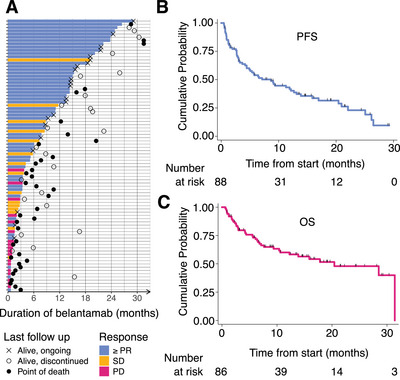
Survival data. (A) PFS and OS curves for the cohort. (B) Swimmer plot, indicating treatment status at last follow‐up. Note the lengthy DoR.

Median follow‐up was 16.1 months (95% CI, 13.5–19.1) by the Kaplan–Meier method. Median PFS was 8.7 months (4.4–12.6), and median OS was 20.4 months (10.4 to incalculable), as shown in Figure 4. The swimmer plot, also shown in Figure 4, shows the distribution of the DoR, where the median was 15.8 months (8.7–25.2). One patient experienced SD for over a year.

### 3.4 Adverse Events

AEs affecting at least 10% of patients are listed in Table 3; a complete table of toxicities is available in Table S1. Of the 85 patients for whom toxicity data were available, 71/85 (84%) experienced toxicity of any grade. By far the most common toxicity was keratopathy (48/85, 56%), with 8/85 (9%) experiencing Grade 3 severity. Altogether, 56/85 patients (66%) experienced ophthalmological symptoms reported as visual blurring, dry eye, blepharitis and eye pain in addition to objective keratopathy findings. Thrombocytopenia was quite frequent (17/85, 20%) and often severe (≥ G3 in 10/85, 12%). The most severe toxicities were chest infections and, specifically, COVID, where the latter was responsible for two deaths. Reduced eGFR did not appear to predict a worse toxicity profile: patients with eGFR < 30 mL/min/1.73 m2 experienced no Grades 4 or 5 AEs, though this was a very small subcohort with only seven patients (see Table S2 for a breakdown of toxicity by renal category).

**TABLE 3:** Adverse events summary noted in at least 10% of patients.

Toxicity (*n* = 85)	Any Grade (%)	At least G3 (%)
Keratopathy	48 (56)	8 (9)
Thrombocytopenia	17 (20)	10 (12)
Chest infection	14 (16)	9 (11)
Blurred vision	14 (16)	3 (4)
Dry eye	12 (14)	0 (0)
Fever	9 (11)	3 (4)

Toxicity led to dose reduction or delay in 35/85 patients (41%); this was not associated with a loss of efficacy–instead, patients seeing a dose deviation were 8.6× more likely to be responders (OR calculated by Fisher's exact test, *p* < 0.0001), presumably because non‐responders have fewer cycles of therapy, so less opportunity for a deviation to occur.

### 3.5 Ocular Toxicity

Owing to the prevalence of ocular toxicity and the understandable concern this generates amongst patients and clinicians, we attempted to gain some granular insight into its impact and the factors that predict it.

**FIGURE 5:**
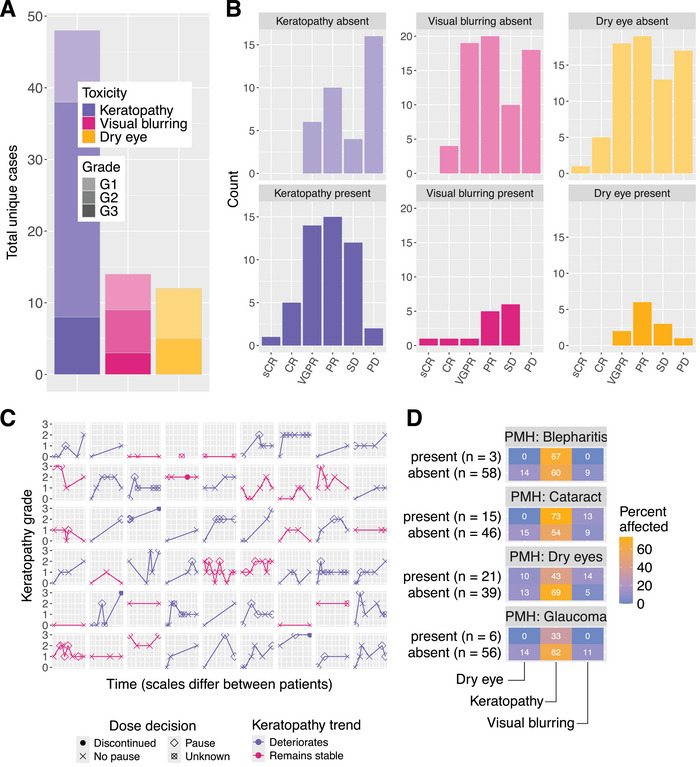
Ocular toxicity. (A) Breakdown of toxicities, categorised following the DREAMM‐2 analysis. (B) No obvious association between ocular toxicity and the distribution of response. (C) Trajectories of KVA grade over time where serial ophthalmological data is available. Each plot represents a patient. Purple trend line—KVA grade is stable/improves, and pink—KVA deteriorates. Each dot represents decision‐making at each assessment. Note five patients discontinued therapy. (D) Heatmap showing the presence or absence of four separate underlying ocular conditions has no bearing on the risk of developing one of the three ocular toxicities. Values represent the percent affected by keratopathy. KVA, keratopathy and visual acuity scale.

Most ocular toxicity was coded in our cohort as keratopathy, visual blurring or dry eye as per categorisation in DREAMM‐2 [27] (Figure 5A). Ophthalmological records were used to characterise the trajectory of ocular toxicity; 33 of the 54 patients with serial data available experienced a worsening of their ocular health during treatment, as evaluated by the KVA or CTCAE grade if KVA was not available (Figure 5C). A total of 42 patients experienced at least one Grade 2 KVA event, and 29 of those—on at least one occasion—did not pause treatment despite this result. The presence of any of the three ocular events did not predict response (Fisher exact test, *p* values of 0.12, 1.00 and 0.76, respectively, and Figure 5B); this may suggest that any attendant dose delay or reduction did not curb successful treatment, but we must be mindful of the confounding effect of the number of cycles received. Critically, of the five patients who discontinued treatment because of side effects < G5, all five did so following keratopathy.

In our cohort, the baseline eye conditions of glaucoma, dry eye, blepharitis or cataract were not significantly predictive of keratopathy, blurred vision or dry eye (Figure 5D).

## 4 Discussion

We present a cohort of patients with relapsed/refractory myeloma from the UK and ROI who have received monotherapy belamaf under the named patient programme. We describe an ORR of 60%, a median PFS of 8.7 and a DoR of 15.8 months at a median follow‐up time of 16.1 months. Belamaf monotherapy was the only BCMA‐targeting therapy for multiply relapsed patients in the United Kingdom and Ireland outside of clinical trials and thus represented an important option in late‐line disease. Perhaps all the more so, given it could be administered entirely in the outpatient setting. Belamaf monotherapy is no longer available globally, but we believe this study remains relevant given that belamaf use, as part of combination therapy, is likely to be a significant proportion of relapsed myeloma therapy [32].

This dataset is outlying relative to other real‐world studies and the landmark DREAMM‐2 trial owing to a high ORR and represents an opportunity to gain insight into the predictors of belamaf response as to what sets the UK and Irish cohort apart from others.

We are not aware of a selection bias in study design because centres were encouraged to submit all patients who had received the drug. Responses were judged by individual contributors using the IMWG criteria and were not independently verified, which could potentially bias the response descriptions—though it seems likely the same shortcoming applies to other retrospective analyses, so this may not provide a sufficient explanation for higher ORR.

Compared to DREAMM‐2, our cohort has a similar age and sex profile; perhaps importantly, our patients were less extensively pretreated (prior lines of therapy 5 vs. 6 in DREAMM‐2, with only 86% vs. 100% triple‐class exposed), and in our group, ISS scores were lower but high‐risk cytogenetics more prevalent. When compared with all published studies as a whole, however, there is no significant heterogeneity between our cohort and others regarding median age, previous autograft, median number of prior lines, percentage high risk and ISS grades. We could hypothesise that triple‐class refractoriness is less likely in our cohort due to fixed‐duration bortezomib and relatively low usage of carfilzomib, but these and other features could not be assessed owing to the necessarily brief publication formats of many of the reports and the consequent lack of data granularity.

From studying our cohort only, the single factor we can find that predicts positive outcomes is early exposure to cyclophosphamide. It is unclear how informative a signal this is—it could simply represent a statistical fluke that would be lost if we could better control for disease risk and other confounders. A putative biological mechanism could involve neoantigen generation, but it is hard to see how plausible this is, given cyclophosphamide exposure substantially predates belamaf's start date, and the effect would have to persist through multiple alternative lines of therapy.

Toxicity in our cohort represents another potential learning point. Given inevitable under‐reporting in this retrospective effort, our figures match relatively well with the DREAMM‐2 cohort, though there is a disparity in the frequency of respiratory infections where we saw a comparatively high number (16% vs. 5%), including two COVID‐related deaths. Discontinuation owing to toxicity was a relatively rare event (5/85 where toxicity data was available), but in all cases, discontinuation followed from the keratopathy, and in patients who were experiencing a meaningful response (1× sCR, 2× VGPR, 2× PR). This speaks to the importance of strategies to mitigate keratopathy—such as reduced doses or longer dosing intervals [33, 34]. It is of interest that our discontinuation rate was similar to the DREAMM‐2 cohort (10% vs. 12.5%, respectively) despite the frequent administration of belamaf in the context of G2 or higher severity, which is at odds with the recommendation of the KVA system. This more lenient dosing strategy might suggest that patient symptomatology was the dominant decider of redosing rather than objective ophthalmological findings.

Our dataset is limited by its reliance on the standard patient record, which leads to a relatively high incidence of missing data; this problem is then compounded by incomplete submission in some domains of our data collection tool. Specifically, we acknowledge the lack of data on prior therapy refractoriness limits the interpretation of our outlying results, though the prerequisite for starting belamaf monotherapy is refractoriness to lenalidomide, daratumumab and PIs, and so it may be reasonable to assume that this applied to most patients.

Once fully explored—in particular, with regards to prior treatment and triple‐class refractoriness—our outlying ORR may additionally provide an insight into the mechanism of belamaf response and how best to sequence this potentially highly valuable therapy: the substantial median DoR suggests that converting non‐responders to responders could have a powerful impact clinically. Fundamentally, we hope these results will inform decision‐making for myeloma physicians and their patients in the UK, Ireland and beyond. Further work is needed to explore the sensitivity to BCMA ADC and the resistance mechanisms to this agent to better select and sequence BCMA‐targeted therapies.

## Conflicts of Interest

Karthik Ramasamy: GSK Ad board; Honoraria; Research funding (institutional); Rakesh Popat: GSK Consultancy; Honoraria; Research funding (institutional). The other authors declare no conflicts of interest.

## Supporting information



Supporting Information

Supporting Information

## Data Availability

Please contact the corresponding author to discuss sharing of data pertaining to this study.
