# Supplementary material for: Belantamab Mafodotin Monotherapy for Multiply‐Relapsed Myeloma: A Retrospective Study From the United Kingdom and the Republic of Ireland
Source: EJHaem. 2025 Apr 30;6(3):e70039. doi: 10.1002/jha2.70039 (PMC12042999; doi:10.1002/jha2.70039)
Supplement: Supplementary file 1 — Supporting Information [file JHA2-6-e70039-s002.docx]

**Supplementary Table 1**

| Toxicity (n = 85) | G1 | | G2 | | G3 | | G4 | | G5 | | Any Grade | |
| --- | --- | --- | --- | --- | --- | --- | --- | --- | --- | --- | --- | --- |
| Keratopathy | 10 | (12) | 30 | (35) | 8 | (9) | 0 | (0) | 0 | (0) | 48 | (56) |
| Thrombocytopenia | 2 | (2) | 5 | (6) | 9 | (11) | 1 | (1) | 0 | (0) | 17 | (20) |
| Chest infection | 2 | (2) | 3 | (4) | 8 | (9) | 1 | (1) | 0 | (0) | 14 | (16) |
| Visual blurring | 5 | (6) | 6 | (7) | 3 | (4) | 0 | (0) | 0 | (0) | 14 | (16) |
| Dry eye | 7 | (8) | 5 | (6) | 0 | (0) | 0 | (0) | 0 | (0) | 12 | (14) |
| Fever | 3 | (4) | 3 | (4) | 3 | (4) | 0 | (0) | 0 | (0) | 9 | (11) |
| COVID | 1 | (1) | 2 | (2) | 1 | (1) | 0 | (0) | 2 | (2) | 6 | (7) |
| Neutropenia | 2 | (2) | 0 | (0) | 4 | (5) | 0 | (0) | 0 | (0) | 6 | (7) |
| Anaemia | 2 | (2) | 0 | (0) | 3 | (4) | 0 | (0) | 0 | (0) | 5 | (6) |
| LFT derangement | 3 | (4) | 1 | (1) | 1 | (1) | 0 | (0) | 0 | (0) | 5 | (6) |
| Fatigue | 3 | (4) | 1 | (1) | 0 | (0) | 0 | (0) | 0 | (0) | 4 | (5) |
| Nausea | 3 | (4) | 1 | (1) | 0 | (0) | 0 | (0) | 0 | (0) | 4 | (5) |
| Infusion reaction | 2 | (2) | 0 | (0) | 1 | (1) | 0 | (0) | 0 | (0) | 3 | (4) |
| Abdominal pain | 1 | (1) | 1 | (1) | 0 | (0) | 0 | (0) | 0 | (0) | 2 | (2) |
| AKI | 1 | (1) | 0 | (0) | 1 | (1) | 0 | (0) | 0 | (0) | 2 | (2) |
| Bacteremia | 0 | (0) | 0 | (0) | 2 | (2) | 0 | (0) | 0 | (0) | 2 | (2) |
| Bone pain | 0 | (0) | 2 | (2) | 0 | (0) | 0 | (0) | 0 | (0) | 2 | (2) |
| Cough | 2 | (2) | 0 | (0) | 0 | (0) | 0 | (0) | 0 | (0) | 2 | (2) |
| Diarrhoea | 1 | (1) | 0 | (0) | 1 | (1) | 0 | (0) | 0 | (0) | 2 | (2) |
| Hypotension | 1 | (1) | 0 | (0) | 1 | (1) | 0 | (0) | 0 | (0) | 2 | (2) |
| Myalgia | 1 | (1) | 1 | (1) | 0 | (0) | 0 | (0) | 0 | (0) | 2 | (2) |
| URT infection | 0 | (0) | 1 | (1) | 1 | (1) | 0 | (0) | 0 | (0) | 2 | (2) |
| UTI | 0 | (0) | 0 | (0) | 2 | (2) | 0 | (0) | 0 | (0) | 2 | (2) |
| Anorexia | 0 | (0) | 1 | (1) | 0 | (0) | 0 | (0) | 0 | (0) | 1 | (1) |
| Bleeding | 0 | (0) | 0 | (0) | 1 | (1) | 0 | (0) | 0 | (0) | 1 | (1) |
| Blepharitis | 1 | (1) | 0 | (0) | 0 | (0) | 0 | (0) | 0 | (0) | 1 | (1) |
| Enterocolitis | 0 | (0) | 0 | (0) | 1 | (1) | 0 | (0) | 0 | (0) | 1 | (1) |
| Epistaxis | 1 | (1) | 0 | (0) | 0 | (0) | 0 | (0) | 0 | (0) | 1 | (1) |
| Fracture | 0 | (0) | 1 | (1) | 0 | (0) | 0 | (0) | 0 | (0) | 1 | (1) |
| Headache | 1 | (1) | 0 | (0) | 0 | (0) | 0 | (0) | 0 | (0) | 1 | (1) |
| Hyponatraemia | 0 | (0) | 1 | (1) | 0 | (0) | 0 | (0) | 0 | (0) | 1 | (1) |
| Insomnia | 1 | (1) | 0 | (0) | 0 | (0) | 0 | (0) | 0 | (0) | 1 | (1) |
| Neutropenic sepsis | 0 | (0) | 0 | (0) | 1 | (1) | 0 | (0) | 0 | (0) | 1 | (1) |
| Chest pain (non-cardiac) | 0 | (0) | 1 | (1) | 0 | (0) | 0 | (0) | 0 | (0) | 1 | (1) |
| Oral infection | 0 | (0) | 1 | (1) | 0 | (0) | 0 | (0) | 0 | (0) | 1 | (1) |
| Pulmonary oedema | 0 | (0) | 1 | (1) | 0 | (0) | 0 | (0) | 0 | (0) | 1 | (1) |
| PE | 0 | (0) | 0 | (0) | 1 | (1) | 0 | (0) | 0 | (0) | 1 | (1) |
| Rash | 0 | (0) | 1 | (1) | 0 | (0) | 0 | (0) | 0 | (0) | 1 | (1) |
| Skin infection | 0 | (0) | 1 | (1) | 0 | (0) | 0 | (0) | 0 | (0) | 1 | (1) |
| Thromboembolism | 0 | (0) | 0 | (0) | 1 | (1) | 0 | (0) | 0 | (0) | 1 | (1) |

**Supplementary Table 2**

**eGFR ≥ 60 ml/min/1.73m^2^**

| Toxicity | G1 | | G2 | | G3 | | G4 | | G5 | | Any Grade | ≥ G3 |
| --- | --- | --- | --- | --- | --- | --- | --- | --- | --- | --- | --- | --- |
| keratopathy | 6 | (7) | 10 | (12) | 3 | (4) | 0 | (0) | 0 | (0) | 19 | 3 |
| chestinfection | 2 | (2) | 2 | (2) | 4 | (5) | 1 | (1) | 0 | (0) | 9 | 5 |
| dryeye | 5 | (6) | 0 | (0) | 0 | (0) | 0 | (0) | 0 | (0) | 5 | 0 |
| thrombocytopenia | 0 | (0) | 1 | (1) | 4 | (5) | 0 | (0) | 0 | (0) | 5 | 4 |
| visblur | 3 | (4) | 2 | (2) | 0 | (0) | 0 | (0) | 0 | (0) | 5 | 0 |
| fever | 1 | (1) | 3 | (4) | 0 | (0) | 0 | (0) | 0 | (0) | 4 | 0 |
| COVID | 1 | (1) | 0 | (0) | 1 | (1) | 0 | (0) | 1 | (1) | 3 | 2 |
| anaemia | 1 | (1) | 0 | (0) | 2 | (2) | 0 | (0) | 0 | (0) | 3 | 2 |
| fatigue | 2 | (2) | 1 | (1) | 0 | (0) | 0 | (0) | 0 | (0) | 3 | 0 |
| neutropenia | 1 | (1) | 0 | (0) | 1 | (1) | 0 | (0) | 0 | (0) | 2 | 1 |
| abdopain | 1 | (1) | 0 | (0) | 0 | (0) | 0 | (0) | 0 | (0) | 1 | 0 |
| bacteremia | 0 | (0) | 0 | (0) | 1 | (1) | 0 | (0) | 0 | (0) | 1 | 1 |
| fracture | 0 | (0) | 1 | (1) | 0 | (0) | 0 | (0) | 0 | (0) | 1 | 0 |
| hyponatraemia | 0 | (0) | 1 | (1) | 0 | (0) | 0 | (0) | 0 | (0) | 1 | 0 |
| hypotension | 1 | (1) | 0 | (0) | 0 | (0) | 0 | (0) | 0 | (0) | 1 | 0 |
| infusion | 0 | (0) | 0 | (0) | 1 | (1) | 0 | (0) | 0 | (0) | 1 | 1 |
| lft | 1 | (1) | 0 | (0) | 0 | (0) | 0 | (0) | 0 | (0) | 1 | 0 |
| myalgia | 1 | (1) | 0 | (0) | 0 | (0) | 0 | (0) | 0 | (0) | 1 | 0 |
| nausea | 1 | (1) | 0 | (0) | 0 | (0) | 0 | (0) | 0 | (0) | 1 | 0 |
| oralinfection | 0 | (0) | 1 | (1) | 0 | (0) | 0 | (0) | 0 | (0) | 1 | 0 |
| pulmonaryembolism | 0 | (0) | 0 | (0) | 1 | (1) | 0 | (0) | 0 | (0) | 1 | 1 |
| 0 | 0 | (0) | 0 | (0) | 0 | (0) | 0 | (0) | 0 | (0) | 0 | 0 |

**eGFR 30 - 60 ml/min/1.73m^2^**

| Toxicity | G1 | | G2 | | G3 | | G4 | | G5 | | Any Grade | ≥ G3 |
| --- | --- | --- | --- | --- | --- | --- | --- | --- | --- | --- | --- | --- |
| keratopathy | 1 | [1] | 8 | [9] | 1 | [1] | 0 | [0] | 0 | [0] | 10 | 1 |
| chestinfection | 0 | [0] | 0 | [0] | 2 | [2] | 0 | [0] | 0 | [0] | 2 | 2 |
| diarrhoea | 1 | [1] | 0 | [0] | 1 | [1] | 0 | [0] | 0 | [0] | 2 | 1 |
| fever | 0 | [0] | 0 | [0] | 2 | [2] | 0 | [0] | 0 | [0] | 2 | 2 |
| COVID | 0 | [0] | 0 | [0] | 0 | [0] | 0 | [0] | 1 | [1] | 1 | 1 |
| aki | 0 | [0] | 0 | [0] | 1 | [1] | 0 | [0] | 0 | [0] | 1 | 1 |
| bleeding | 0 | [0] | 0 | [0] | 1 | [1] | 0 | [0] | 0 | [0] | 1 | 1 |
| dryeye | 0 | [0] | 1 | [1] | 0 | [0] | 0 | [0] | 0 | [0] | 1 | 0 |
| hypotension | 0 | [0] | 0 | [0] | 1 | [1] | 0 | [0] | 0 | [0] | 1 | 1 |
| insomnia | 1 | [1] | 0 | [0] | 0 | [0] | 0 | [0] | 0 | [0] | 1 | 0 |
| nausea | 0 | [0] | 1 | [1] | 0 | [0] | 0 | [0] | 0 | [0] | 1 | 0 |
| neutsepsis | 0 | [0] | 0 | [0] | 1 | [1] | 0 | [0] | 0 | [0] | 1 | 1 |
| pulmoedema | 0 | [0] | 1 | [1] | 0 | [0] | 0 | [0] | 0 | [0] | 1 | 0 |
| thrombocytopenia | 0 | [0] | 1 | [1] | 0 | [0] | 0 | [0] | 0 | [0] | 1 | 0 |
| visblur | 0 | [0] | 0 | [0] | 1 | [1] | 0 | [0] | 0 | [0] | 1 | 1 |
| 0 | 0 | [0] | 0 | [0] | 0 | [0] | 0 | [0] | 0 | [0] | 0 | 0 |

**eGFR < 30 ml/min/1.73m^2^**

| Toxicity | G1 | | G2 | | G3 | | G4 | | G5 | | Any Grade | ≥ G3 |
| --- | --- | --- | --- | --- | --- | --- | --- | --- | --- | --- | --- | --- |
| abdopain | 0 | [0] | 1 | [1] | 0 | [0] | 0 | [0] | 0 | [0] | 1 | 0 |
| anaemia | 0 | [0] | 0 | [0] | 1 | [1] | 0 | [0] | 0 | [0] | 1 | 1 |
| anorexia | 0 | [0] | 1 | [1] | 0 | [0] | 0 | [0] | 0 | [0] | 1 | 0 |
| fever | 1 | [1] | 0 | [0] | 0 | [0] | 0 | [0] | 0 | [0] | 1 | 0 |
| thrombocytopenia | 0 | [0] | 0 | [0] | 1 | [1] | 0 | [0] | 0 | [0] | 1 | 1 |
| utinfection | 0 | [0] | 0 | [0] | 1 | [1] | 0 | [0] | 0 | [0] | 1 | 1 |

**Supp Fig 1**

**Supp Fig 2 – Toxicity events causing dose reduction or delay**
