# Supplementary material for: Belantamab Mafodotin Monotherapy for Multiply‐Relapsed Myeloma: A Retrospective Study From the United Kingdom and the Republic of Ireland
Source: EJHaem. 2025 Apr 30;6(3):e70039. doi: 10.1002/jha2.70039 (PMC12042999; doi:10.1002/jha2.70039)
Supplement: Supplementary file 2 — Supporting Information [file JHA2-6-e70039-s001.pdf]

Protocol ID: ____________________

Study Name: ____________________

Site: ____________________

Event Name: ____________________

Event Date: ____________________

Study Subject ID:___________

Interviewer Name:___________

Interview Date:___________

1-Proj-MMY-98-Baseline-Patient-Characteristics - 1

Section Title: Disease

Myeloma Diagnosis Date:

Performance Status
Date:

ECOG

- ◯ Please enter
- ◯ 0 - Fully active. Able to carry on all pre-disease performance without restriction
- ◯ 1 - Restricted in physically strenuous activity but ambulatory and able to carry out work of a light or sedentary nature.
- ◯ 2 - Ambulatory and capable of all selfcare but unable to carry out any work activities. Up and about more than 50% of waking hours
- ◯ 3 - Capable of only limited selfcare; confined to bed or chair more than 50% of waking hours
- ◯ 4 - Completely disabled. Cannot carry on any selfcare. Totally confined to bed or chair
- ◯ 5 - Dead
- ◯ 6 - Not Known

Protocol ID: ____________________
Study Name: ____________________
Site: ____________________
Event Name: ____________________
Event Date: ____________________

Study Subject ID:___________
Interviewer Name:___________
Interview Date:___________

Section Title: Comorbidities

Comorbidities

Age:
- Select
- <50 _tmplitem="7" years
- 50-59 years
- 60-69 years
- 70-79 years
- ≥80 years

Myocardial Infarction:
- Select
- No
- Yes

History of definite or probable MI (EKG changes and/or enzyme changes)

Congestive Heart Failure:
- Select
- No
- Yes

Exertional or paroxysmal nocturnal dyspnea and has responded to digitalis, diuretics, or afterload reducing agents

Peripheral Vascular Disease:
- Select
- No
- Yes

Intermittent claudication or past bypass for chronic arterial insufficiency,
history of gangrene or acute arterial insufficiency, or untreated thoracic or abdominal aneurysm (≥6 cm)

Cerebrovascular Accident / Transient Ischaemic Attack:
- Select
- No
- Yes

Dementia:
- Select
- No
- Yes

Chronic cognitive deficit

Chronic Obstructive Pulmonary Disease:

- Select
- No
- Yes

Connective Tissue Disease:

- Select
- No
- Yes

Peptic Ulcer Disease:

- Select
- No
- Yes

Any history of treatment for ulcer disease or history of ulcer bleeding

Liver Disease:

- Select
- None
- Mild
- Moderate to Severe

Severe = cirrhosis and portal hypertension with variceal bleeding history, moderate = cirrhosis and portal hypertension but no variceal bleeding history, mild = chronic hepatitis (or cirrhosis without portal hypertension)

Diabetes Millitus:

- Select
- None or diet-controlled
- Uncomplicated
- End-organ damage

Hemiplegia:

- Select
- No
- Yes

Moderate to Severe Chronic Kidney Disease:

- Select
- No
- Yes

Severe = on dialysis, status post kidney transplant, uremia, moderate = creatinine >3 mg/dL (0.27 mmol/L)

Solid Tumour:

- Select
- None
- Localised
- Metastatic

Leukaemia:

- ◯ Select
- ◯ No
- ◯ Yes

Lymphoma:

- ◯ Select
- ◯ No
- ◯ Yes

AIDS:

- ◯ Select
- ◯ No
- ◯ Yes

Calculate the score via: https://www.mdcalc.com/charlson-comorbidity-index-cci

Charlson Score:

Charlson Category:

- ◯ Select
- ◯ 0-2
- ◯ 3-4
- ◯ >=5

Protocol ID: ____________________

Study Name: ____________________

Site: ____________________

Event Name: ____________________

Event Date: ____________________

Study Subject ID:____________

Interviewer Name:____________

Interview Date:____________

Section Title: Lab

Bloods Date:

Creatinine: (micromol/L)

e-GRF:

e-GRF Category:
- ◯ Select
- ◯ ≥60
- ◯ 30-59
- ◯ <30

Anaemia:
- ◯ Select
- ◯ No
- ◯ Yes

Lymphopenia:
- ◯ Select
- ◯ No
- ◯ Yes

Hypercalcaemia:
- ◯ Select
- ◯ No
- ◯ Yes

Neutropenia:
- ◯ Select
- ◯ No
- ◯ Yes

Thrombocytopenia:
- ◯ Select
- ◯ No
- ◯ Yes

Protocol ID: ____________________

Study Name: ____________________

Site: ____________________

Event Name: ____________________

Event Date: ____________________

Study Subject ID:____________

Interviewer Name:____________

Interview Date:____________

Section Title: Eye History

Glaucoma:

- ◯ Select
- ◯ No
- ◯ Yes
- ◯ Not Known

Cataracts:

- ◯ Select
- ◯ No
- ◯ Yes
- ◯ Not Known

Blepharitis:

- ◯ Select
- ◯ No
- ◯ Yes
- ◯ Not Known

Dry Eyes:

- ◯ Select
- ◯ No
- ◯ Yes
- ◯ Not Known

Protocol ID: ____________________

Study Name: ____________________

Site: ____________________

Event Name: ____________________

Event Date: ____________________

Study Subject ID:____________

Interviewer Name:____________

Interview Date:____________

2-Proj-MMY-98-Baseline-Occular-Assessments - 1

Section Title: Ocular Examination

Left Eye

Snellen metric acuity (left eye):

Right Eye

Snellen metric acuity (right eye):

Left Eye

Intraocular Pressure (left eye):

Right Eye

Intraocular Pressure (right eye):

Left Eye

Conjunctival Injection (left eye):

- ○ Select
- ○ Normal/None
- ○ Prominence of conjunctival vessels without ciliary inj - episcleral inj - no haemorrhage
- ○ Obvious dilation of conjunctival vessels - may have mild ciliary inj - no episcleral inj - no haemorrhage
- ○ Severe inj - ciliary inj - may have subconjunctival haemorrhage - or episcleral inj

Chemosis (left eye):

- ○ Select
- ○ Normal/none
- ○ Slight oedema - localised or not diffuse
- ○ Diffuse oedema but clear serous fluid without significant prolapse over the lower lid
- ○ Diffuse oedema with hazy subconjunctival fluid and significant prolapse of conjunctiva over the lower lid

Right Eye

Conjunctival Injection (right eye):

- Select
- Normal/None
- Prominence of conjunctival vessels without ciliary inj - episcleral inj - no haemorrhage
- Obvious dilation of conjunctival vessels - may have mild ciliary inj - no episcleral inj - no haemorrhage
- Severe inj - ciliary inj - may have subconjunctival haemorrhage - or episcleral inj

Chemosis (right eye):

- Select
- Normal/none
- Slight oedema - localised or not diffuse
- Diffuse oedema but clear serous fluid without significant prolapse over the lower lid
- Diffuse oedema with hazy subconjunctival fluid and significant prolapse of conjunctiva over the lower lid

Left Eye

Corneal Epithelium (left eye):

- Select
- Normal
- Abnormal

Punctate Keratopathy (left eye):

- Select
- Normal
- Mild punctate keratopathy
- Moderate punctate keratopathy
- Severe punctate keratopathy

Epithelial Odema (left eye):

- Select
- Normal
- Suble epithelial haze
- Mild patchy microcytic changes
- Diffuse microcytic changes

Stroma (left eye):

- Select
- Normal
- Abnormal

Stroma Active Opacity (left eye):

- Select
- Mild active opacity
- Moderate active opacity
- Severe active opacity

Stroma Active Oedema (left eye):
- Select
- Trace
- 1+
- 2+
- 3+
- 4+

Endothelium (left eye):
- Select
- Normal
- Guttata
- Descemet's folds
- Other (please specify)

Endothelium (left eye) Comments:

Right Eye

Corneal Epithelium (right eye):
- Select
- Normal
- Abnormal

Punctate Keratopathy (right eye):
- Select
- Normal
- Mild punctate keratopathy
- Moderate punctate keratopathy
- Severe punctate keratopathy

Epithelial Odema (right eye):
- Select
- Normal
- Suble epithelial haze
- Mild patchy microcytic changes
- Diffuse microcytic changes

Stroma (right eye):
- Select
- Normal
- Abnormal

Stroma Active Opacity (right eye):
- ◯ Select
- ◯ Mild active opacity
- ◯ Moderate active opacity
- ◯ Severe active opacity

Stroma Active Oedema (right eye):
- ◯ Select
- ◯ Trace
- ◯ 1+
- ◯ 2+
- ◯ 3+
- ◯ 4+

Endothelium (right eye):
- ◯ Select
- ◯ Normal
- ◯ Guttata
- ◯ Descemet's folds
- ◯ Other (please specify)

Endothelium (left eye) Comments:

Protocol ID: ____________________

Study Name: ____________________

Site: ____________________

Event Name: ____________________

Event Date: ____________________

Study Subject ID:____________

Interviewer Name:____________

Interview Date:____________

3-Proj-MMY-98-Baseline-Disease-Characteristics - 1

Section Title: Disease

Disease

MM Subtype:

- ◯ Please enter
- ◯ LC
- ◯ IgG
- ◯ IgA
- ◯ IgM
- ◯ IgD
- ◯ Oligosecretory
- ◯ No-Sec

Amyloidosis?:

- ◯ Select
- ◯ No
- ◯ Yes

Plasma cell leukaemia?:

- ◯ Select
- ◯ No
- ◯ Yes

Extramedullary disease present?:

- ◯ Select
- ◯ No
- ◯ Yes

Protocol ID: ____________________

Study Name: ____________________

Site: ____________________

Event Name: ____________________

Event Date: ____________________

Study Subject ID:____________

Interviewer Name:____________

Interview Date:____________

Section Title: Lab

Lab

Bloods Date:

Albumin: (g/L)

Albumin Category
- ◯ Select
- ◯ <35g/l
- ◯ ≥35g/l

B2M: (mg/L)

B2M Category:
- ◯ Select
- ◯ <3.5
- ◯ 3.5-5.4
- ◯ >5.4

LDH: (u/L)

LDH Elevated
- ◯ Select
- ◯ No
- ◯ Yes

Protocol ID: ____________________

Study Name: ____________________

Site: ____________________

Event Name: ____________________

Event Date: ____________________

Study Subject ID:____________

Interviewer Name:____________

Interview Date:____________

Section Title: Cytogenetics

NB. All known cytogenetic abnormalities

Bone Marrow Date:

Percentage Plasma Cells: (%)

Cytogenetics Cytogenetic Risk:
- ◯ Select
- ◯ 0 - Standard Risk
- ◯ 1 - High Risk
- ◯ 3 - Unknown

Cytogenetics Cytogenetic Abnormalities:
- ☐ t(4;14)
- ☐ t(14;16)
- ☐ t(14;20)
- ☐ del(17p)
- ☐ 1p loss
- ☐ 1qgain/amp
- ☐ del 13q
- ☐ Hypodiploidy
- ☐ Other (specify)
- ☐ Unknown

Cytogenetics Other Abnormalities:

Protocol ID: ____________________

Study Name: ____________________

Site: ____________________

Event Name: ____________________

Event Date: ____________________

Study Subject ID:____________

Interviewer Name:____________

Interview Date:____________

Section Title: Staging

Staging
You can calculate R-ISS staging via: https://www.mdcalc.com/revised-multiple-myeloma-international-staging-system-r-iss

ISS Staging

- Select
- Stage I
- Stage II
- Stage III
- Not Known

R-ISS Staging

- Select
- Stage I
- Stage II
- Stage III
- Not Known

Protocol ID: ____________________

Study Name: ____________________

Site: ____________________

Event Name: ____________________

Event Date: ____________________

Study Subject ID:____________

Interviewer Name:____________

Interview Date:____________

Section Title: Symptoms

Symptoms at time of starting belantamab

Fatigue?
- ◯ Select
- ◯ No
- ◯ Yes
- ◯ NK

Pain?
- ◯ Select
- ◯ No
- ◯ Yes
- ◯ NK

Protocol ID: ____________________
Study Name: ____________________
Site: ____________________
Event Name: ____________________
Event Date: ____________________

Study Subject ID:____________
Interviewer Name:____________
Interview Date:____________

4-Proj-MMY-98-Belantamab-Treatment - 1

Section Title: Belantanab

Belanatanab

Belantanab Start Date:

Belantanab End Date: If treatment is ongoing, leave blank

Belantanab Number of Cycles Received:

Belantanab Dosing

Preservative-free Eyedrops:
- Select
- No
- Yes
- Not known

Cooling Eye Mask:
- Select
- No
- Yes
- Not known

Bandage Contact Lens:
- Select
- No
- Yes
- Not known

Starting Dose:
- Select
- 2.5mg/kg
- 1.9mg/kg

Dose Reduction:

- Select
- No
- Yes
- N/A

New (reduced) Dose:
- Select
- 1.9mg/kg
- Other lower dose

New (reduced) Dose Comment:

Reduction in Frequency?
- Select
- 6-weekly
- Other

Reduction Reason:

Prophylaxis

PCP Prophylaxis?:
- Select
- No
- Yes

Antiviral Prophylaxis?:
- Select
- No
- Yes

Fungal Prophylaxis?:
- Select
- No
- Yes

Best response achieved to date

MM Response
- Select
- CR
- sCR
- VGPR
- PR
- Minor Response or Stable Disease
- PD
- Not Known

MM Response Date:

Relapse

Relapse

- Select
- No
- Yes
- Not known

Relapse Comment: Please note biochemical/clinical,radiological replase -- whichever occurred first

Date of Relapse:

Protocol ID: ____________________

Study Name: ____________________

Site: ____________________

Event Name: ____________________

Event Date: ____________________

Study Subject ID:____________

Interviewer Name:____________

Interview Date:____________

Section Title: Prior Treatment

Prior Treatment

Prior Treatment	Treatment Line Number:	Treatment Line Description:	Treatment Date Start:	Treatment Date End:	Number of Cycles:	Best Response:	Relapse Date:
						◯ Select<br>◯ CR<br>◯ sCR<br>◯ VGPR<br>◯ PR<br>◯ Minor Response or Stable Disease<br>◯ PD<br>◯ Not Known	

Protocol ID: ____________________

Study Name: ____________________

Site: ____________________

Event Name: ____________________

Event Date: ____________________

Study Subject ID:____________

Interviewer Name:____________

Interview Date:____________

Section Title: Prior Treatment

Prior Transplant?

- ◯ Select
- ◯ No
- ◯ Yes

Protocol ID: ____________________

Study Name: ____________________

Site: ____________________

Event Name: ____________________

Event Date: ____________________

Study Subject ID:___________

Interviewer Name:___________

Interview Date:___________

5-Proj-MMY-98-Treatments-Toxicity - 1

Section Title: AEs

AEs

AE Date:	AE Name:	AE Grade	Inpatient Stay Days:	Dose Reduction?	Treatment Discontinued?
		◯ Select<br>◯ 1<br>◯ 2<br>◯ 3<br>◯ 4<br>◯ 5		◯ Select<br>◯ No<br>◯ Yes	◯ Select<br>◯ No<br>◯ Yes

Protocol ID: ____________________

Study Name: ____________________

Site: ____________________

Event Name: ____________________

Event Date: ____________________

Study Subject ID:____________

Interviewer Name:____________

Interview Date:____________

Section Title: Infections

Infections				
**Infection Name:**	**Infection Grade:**	**Inpatient Stay Days:**	**Dose Reduction?**	**Treatment Discontinued?**
	◯ Select<br>◯ 1<br>◯ 2<br>◯ 3<br>◯ 4<br>◯ 5		◯ Select<br>◯ No<br>◯ Yes	◯ Select<br>◯ No<br>◯ Yes

Protocol ID: ____________________

Study Name: ____________________

Site: ____________________

Event Name: ____________________

Event Date: ____________________

Study Subject ID:____________

Interviewer Name:____________

Interview Date:____________

Section Title: Infusion Reactions

Infusion Reactions

Infusion Reaction Date:	Infusion Cycle Number Reaction:	Infusion Grade	Infusion Discontinued Treatement?
		◯ Select<br>◯ 1<br>◯ 2<br>◯ 3<br>◯ 4<br>◯ 5	◯ Select<br>◯ No<br>◯ Yes

Protocol ID: ____________________

Study Name: ____________________

Site: ____________________

Event Name: ____________________

Event Date: ____________________

Study Subject ID:___________

Interviewer Name:___________

Interview Date:___________

Section Title: Supportive Treatment

Supportive Treatment	
**Supportive Treatment Date**	**Supportive Treatment:**
	◯ Select<br>◯ RBC transfusino during treatment<br>◯ Platelet transfusion during treatment<br>◯ Filgrastim during treatment

Protocol ID: ____________________

Study Name: ____________________

Site: ____________________

Event Name: ____________________

Event Date: ____________________

Study Subject ID:____________

Interviewer Name:____________

Interview Date:____________

Section Title: Occular Toxicities

Occular Toxicities

Occular AE Date:	Occular AE Name:	Occular AE Grade:	Occular AE Admission:	Occular AE Admission Days:	Occular AE Reduced Dose:	Occular AE Delay:	Occular AE Discontinued:
		◯ Select<br>◯ 1<br>◯ 2<br>◯ 3<br>◯ 4<br>◯ 5	◯ Select<br>◯ No<br>◯ Yes		◯ Select<br>◯ No<br>◯ Yes	◯ Select<br>◯ No<br>◯ Yes	◯ Select<br>◯ No<br>◯ Yes

Protocol ID: ____________________

Study Name: ____________________

Site: ____________________

Event Name: ____________________

Event Date: ____________________

Study Subject ID:____________

Interviewer Name:____________

Interview Date:____________

6-Proj-MMY-98-Opthalmology-Follow-ups - 1

Section Title: Opthalmology Follow-up

Opthalmology Follow-up					
**Opthalmology Follow-up Number:**	**Opthalmology Follow-up Date:**	**Opthalmology Follow-up Findings:**	**Opthalmology Follow-up Belantanab Cycle:**	**Opthalmology Follow-up Belantanab Delay:**	**Opthalmology Follow-up Belantanab Discontinue:**
				◯ Select<br>◯ No<br>◯ Yes	◯ Select<br>◯ No<br>◯ Yes

Protocol ID: ____________________

Study Name: ____________________

Site: ____________________

Event Name: ____________________

Event Date: ____________________

Study Subject ID:____________

Interviewer Name:____________

Interview Date:____________

7-Proj-MMY-98-Subsequent-Treatments - 1

Section Title: Subsequent Treatment

Subsequent Treatment

Treatment Start Date:	Age at Treatment Start Date:	Treatment End Date:	Treatment Name:	Number of Cycles Received:	Response	MM Response Date:	Relapse
					◯ Select<br>◯ CR<br>◯ sCR<br>◯ VGPR<br>◯ PR<br>◯ Minor Response or Stable Disease<br>◯ PD		

Protocol ID: ____________________

Study Name: ____________________

Site: ____________________

Event Name: ____________________

Event Date: ____________________

Study Subject ID:___________

Interviewer Name:___________

Interview Date:___________

8-Proj-MMY-98-Survival-Data - 1

Section Title: Demographics

Gender: *

- Please enter
- Male
- Female

Age at start of Belantamab: * (years)

Protocol ID: ____________________

Study Name: ____________________

Site: ____________________

Event Name: ____________________

Event Date: ____________________

Study Subject ID:____________

Interviewer Name:____________

Interview Date:____________

Section Title: Vital Status

Last Follow Up: *

Last Follow Up Vital Status *

- ◯ Please enter
- ◯ Alive
- ◯ Dead

Date of Death:

Reason for Death (if applicable): *

- ◯ Please enter if applicable
- ◯ Progressive Disease
- ◯ Treatment related
- ◯ Other (specify)
- ◯ Not known

Reason for Death Description (if applicable):

Death within 30 days of Treatment: *

- ◯ Please enter
- ◯ Yes
- ◯ No
- ◯ NA

Protocol ID: ____________________

Study Name: ____________________

Site: ____________________

Event Name: ____________________

Event Date: ____________________

Study Subject ID:____________

Interviewer Name:____________

Interview Date:____________

Section Title: Symptoms

Symptoms and Wellbeing at Last Follow-up

Fatigue compared to baseline

- ○ Select
- ○ Improvement
- ○ Deterioration
- ○ Same Baseline
- ○ NK

Pain compared to baseline

- ○ Select
- ○ Improvement
- ○ Deterioration
- ○ Same Baseline
- ○ NK

Performance Status Review:

- ○ Select
- ○ Improvement
- ○ Deterioration
- ○ Same Baseline
- ○ NK

Protocol ID: ____________________

Study Name: ____________________

Site: ____________________

Event Name: ____________________

Event Date: ____________________

Study Subject ID:____________

Interviewer Name:____________

Interview Date:____________

Section Title: COVID-19

Vaccinated against COVID-19?

- ◯ Please enter
- ◯ Yes
- ◯ No

Number of COVID-19 vaccine doses received?

Date of last COVID-19 vaccine:

Anti-body Test Performed?

- ◯ Please enter
- ◯ Yes
- ◯ No

Anti-body Test Date:

Anti-bodies Detected?

- ◯ Please enter
- ◯ Yes
- ◯ No
- ◯ NA

Protocol ID: ____________________
Study Name: ____________________
Site: ____________________
Event Name: ____________________
Event Date: ____________________

Study Subject ID:____________
Interviewer Name:____________
Interview Date:____________

9_proj_mmy_98_follow_up - 1

Section Title: Belantamab

Total Cycles Received: * Please enter the cumulative total (i.e. not just those given since the previous data entry point.)

Best Response: *

- ○ Please enter
- ○ sCR
- ○ CR
- ○ VGPR
- ○ PR
- ○ Stable disease
- ○ Progressive Disease
- ○ Unknown

Protocol ID: ____________________

Study Name: ____________________

Site: ____________________

Event Name: ____________________

Event Date: ____________________

Study Subject ID:____________

Interviewer Name:____________

Interview Date:____________

Section Title: Discontinuation

Is belantamab ongoing? *

- ◯ No
- ◯ Yes

If "Yes" please move onto Vital Status tab, If "No" complete the following.

Date of Final Dose:

Why was belantamab discontinued?

- ◯ Please enter
- ◯ Relapse/Progressive Disease
- ◯ Toxicity
- ◯ Non-relapse mortality
- ◯ Other

If relapse/progressive disease when was this confirmed:

If toxicity, what was the adverse event?

Please give grade and adverse event (e.g. G4 keratopathy)

If other, please describe:

Was the next line of treatment started?

- ◯ Please enter
- ◯ No
- ◯ Yes
- ◯ Unknown

If yes, please describe the line(s) of treatment:

Protocol ID: ____________________
Study Name: ____________________
Site: ____________________
Event Name: ____________________
Event Date: ____________________

Study Subject ID:____________
Interviewer Name:____________
Interview Date:____________

Section Title: Vital Status

Date of the most recent follow-up: *

Is the patient currently alive? *

- No
- Yes

If "No" then please complete the following

Death Date:

Death Cause:
